# The Longitudinal Effects of Depression on Academic Performance in Chinese Adolescents via Peer Relationships: The Moderating Effect of Gender and Physical Activity

**DOI:** 10.3390/ijerph20010181

**Published:** 2022-12-23

**Authors:** Yingchen Bi, Minkwon Moon, Myoungjin Shin

**Affiliations:** 1 Department of Leisure Sports, Kangwon National University, Samcheok 25913, Republic of Korea; 2 Department of Physical Education, Seoul National University, Seoul 143701, Republic of Korea

**Keywords:** depression, academic performance, peer relationships, gender, physical activity

## Abstract

This study aims to examine whether there is a difference in the sequential effect of depression on academic performance through peer relationships in Chinese adolescents based on gender and physical activity by using longitudinal data. Data from 7170 people from the China Education Panel Survey (CEPS) were used for analysis. The analysis showed that the indirect effect of depression on academic performance through peer relationships varied depending on gender and physical activity. Specifically, in male students experiencing depression, there was an indirect effect on academic performance through peer relationships in both high and low physical activity groups. In female students experiencing depression, the indirect effect on academic performance through peer relationships was offset if they participated more in physical activity. In other words, for female students participating in physical activity, improvement in peer relationships did not lead to higher academic performance, whereas for male students, improvement in academic performance could be anticipated by improving peer relationships regardless of participation in physical activity. Moreover, since peer relationships play a critical role in improving the academic performance of adolescents experiencing depression, it is important to develop a physical activity or sports program to strengthen peer relationships to improve academic performance.

## 1. Introduction

A recent study in China investigated the mental health status of 4153 adolescents and found that 27.5% of the sample suffered from depression [1]. Adolescence is a period in which a person experiences great psychological and physiological changes and spends more and more time building social relationships with peers rather than with parents [2]. This has a significant impact on the social adjustment of adolescents [3]. Experiences of peer rejection in adolescence cause emotion regulation and sociability issues, some of which are observed after 10 years [4,5,6]. Those who experience low acceptance from peers are known to show not only hindered academic performance [7] but also greater loneliness and depression [8]. Conversely, those who receive high acceptance from peers show better pro-social behavior [9], improved academic performance [10], and are less lonely, indicating a positive effect on various forms of social, academic, and psychological adjustment [11]. Using longitudinal data, this study examines how the relationship between peer relationships, depression, and the academic performance of adolescents vary depending on gender and physical activity.

### 1.1. Depression and Academic Performance

Improving the academic performance of students is one of the fundamental goals of educational planning at the national level, and this may also become the criterion for evaluating the qualitative improvement of education [12]. Chinese society has traditionally valued children’s academic performance and strongly tends to view education as an important channel for improving the socioeconomic status of individuals and households, achieving success, and pursuing a better life [13]. Adolescents transitioning from elementary to middle school face a new learning environment, as well as significant changes in academic content and academic demands. Those having adjustment difficulties experience negative emotions and can develop defense mechanisms, thereby resulting in low academic performance [14].

To summarize existing studies, student depression is one of many factors that negatively affect academic performance [15,16]. In general, students with depression focus on intrusive and irrelevant thoughts, thereby failing to concentrate on continuous cognitive tasks, leading to academic failure [16]. Moreover, students experiencing depression have low academic self-efficacy and academic expectations, as well as high test anxiety in learning, which leads to academic procrastination, thereby deteriorating academic performance [17,18,19]. A longitudinal study on adolescents conducted for six years proved the negative relationship between depression and academic performance [15]. In a meta-analysis examining the relationship between depression and academic performance based on 43 longitudinal studies (a total of 24,557 participants), a negative correlation (r = −0.15) was confirmed between depression and academic performance [20].

### 1.2. Depression, Academic Performance, and Peer Relationship

Peer relationships can be divided into “peer acceptance”, which is the degree to which one is graded or welcomed by peers, and “friendship”, which is a mutual one-on-one relationship between friends. The former is a one-way relationship in which the group is oriented toward the individual and represents the group’s opinion about the individual, whereas the latter is a two-way relationship that refers to the emotional relationship occurring between two individuals [21].

In adolescence, the time spent with peers increases significantly compared to childhood and thus peer relationships have a critical impact on the formation of social relations [22]. In general, depression is one of the main factors inhibiting peer relationships. According to interpersonal theories of depression, individuals who tend to have depression face problems in forming social relations with others [23,24]. In other words, people with depression lack the effort and will to form relationships as they refuse encouragement and comfort from others, and this is exacerbated among adolescents [25].

Students being actively preferred by peers and being popular tends to have a positive effect not only on their emotional adaptability but also on academic performance [26]. Further, the formation of good peer relationships helps build a strong sense of belonging and attachment to school, which leads to high motivation and a good attitude toward learning [27]. Students with high peer acceptance at school show better academic performance than students that are marginalized [26]. Wentzel [28] also observed a positive relationship between the number of peers and peer acceptance with academic performance.

### 1.3. The Role of Moderator Variable: Gender and Physical Activity

The primary goal of school classes is to improve the academic performance of students [29] and many studies have proved the positive relationship between physical activity and academic performance [30,31]. Moreover, exercise stimulates several neuroplastic processes related to depression and increases resilience to physiological stress [32]. A study by Hong [33] conducted among Chinese adolescents found that physical activity was negatively associated with depression. A study on European adolescents also found that a lower frequency of participation in physical activity led to a greater risk of depression and that regular physical activity helped reduce anxiety and depression [34].

Exercising with peers can be a critical determinant that helps form peer relationships. Daniels [35] discovered that sports participation is an effective way to be acknowledged by peers and that, regardless of gender, it helps build positive social relations. Moreover, activities conducted with peers in gym class may lead to friendships by meeting individual social needs and developing a sense of belonging [36].

In general, the sensibility of interpersonal relations varies according to gender. Here, male students tend to gain friendship support through externalization and high companionship through emotional connection, whereas female students more strongly tend to gain the acceptance of others through internalization in self-perception [37]. Furthermore, female students are known to value affection and need more human relationships than male students [37]. A recent longitudinal study examined the relationship between peer relationships and academic performance in male and female students and discovered that, unlike male students, female students showed higher academic performance when they perceived peer relationships more positively [38].

Moreover, differences have been found between male and female students in depression and physical activity. Female students in adolescence show negative self-awareness tendencies toward themselves by using more internal defensive measures than male students in response to external threats. Whereas male students use more external mechanisms, such as anger and aggression, to cope with external threats. Therefore, depressive emotions such as guilt, self-disappointment, and sorrow are more prominent in female students than in male students [39,40].

In general, sports and physical activity have been regarded as tools that emphasize masculine culture and are exclusive to men [41]. Sports participation has different characteristics according to gender. Male students in adolescence are more active in sports than female students, which is a phenomenon that is consistent from early childhood [42]. Jago [43] measured the difference in physical activity between male and female students in adolescents and discovered that male students spend less time sitting down and more time engaging in moderate or high-intensity physical activity than female students. Moreover, male students showed more interest and enjoyed physical activity more and physical activity reduced more noticeably in female students than male students as they grew older [44].

### 1.4. Summary and Research Hypotheses

**Figure 1:**
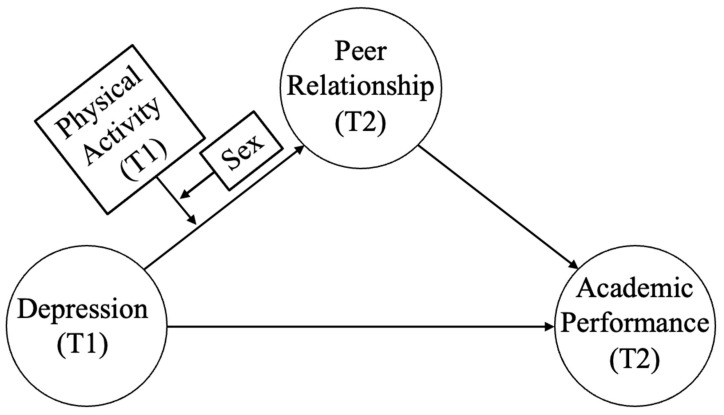
Moderated moderated-mediation model A (T1 represents time point 1, which represents the survey data from 2013; T2 represents time point 2, which represents the survey data from 2014).

**Figure 2:**
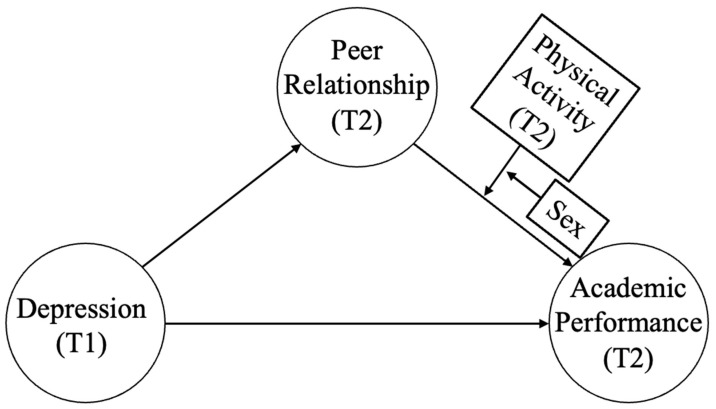
Moderated moderated-mediation model B (T1 represents time point 1, which represents the survey data in 2013; T2 represents time point 2, which represents the survey data in 2014).

Summarily, the previous study shows that depression is a key variable predicting academic performance and, for students with high depression, their depression had a negative effect on their peer relationships, leading to lower academic performance in the mediator model. This relationship between depression, peer relationship, and academic performance may vary depending on gender and physical activity. Thus, to clearly determine the relationship between depression and academic performance through peer relationships, it is desirable to use gender and physical activity as moderator variables. Therefore, this study set the research models as shown in Figure 1 and Figure 2 in which the sequential relationship of depression, peer relationship, and academic performance varied depending on the two moderator variables (physical activity and gender). More specific research hypotheses are as follows.

First, for female students who participate more in physical activity, the indirect effect of depression on academic performance through peer relationships is lower than for female students who participate less in physical activity.

Second, for male students who participate more in physical activity, the indirect effect of depression on academic performance through peer relationships is higher than for male students who participate less in physical activity.

## 2. Materials and Methods

### 2.1. Research Subjects

The China Education Panel Survey (CEPS) initiated a survey project in 2013 targeting 112 schools and 438 classes nationwide. It is the first large-scale longitudinal data survey conducted in China. Out of data from 9949 respondents who participated in the survey for two years in 2013 (Grade 7 (Time 1)) and 2014 (Grade 8 (Time 2)), this study used data from 7170 respondents in the final analysis, excluding that from 2779 respondents who participated in the 2013 survey but not in the 2014 survey or who gave insincere responses. There were 3647 male (50.9%) and 3524 female students (49.1%). The average age of the respondents was 12.5 years (SD = 0.68). Data sources can be found at http://ceps.ruc.edu.cn (accessed on 2 May 2022).

### 2.2. Measurement

#### 2.2.1. Physical Activity (PA)

Daily exercising time (minutes) was coded as follows: 0 min = “1”, 0—less than 30 min = “2”, 30—less than 60 min = “3”, 60—less than 90 min = “4”, 90—less than 120 min = “5”, and 120 min or more = “6”. The value for PA used was calculated by multiplying the daily exercising time coded by the number of exercising days, with higher scores indicating more physical activity in a week.

#### 2.2.2. Academic Performance (AP)

The value for AP used was the mid-term grades for Chinese language, mathematics, and English provided by the surveyed school. This study converted the grades for these three subjects into standardized scores and used the total as the final score.

#### 2.2.3. Perceived Depression (PD)

Perceived depression was measured with a total of five items “Feeling blue,” “Depressed”, “Unhappy”, “Not enjoying”, and “Sad life”. These items were measured on a five-point Likert scale (1 = Never, 5 = Always) (α for 5 items was 0.85).

#### 2.2.4. Perceived Peer Relationship (PPR)

Perceived peer relationship (PPR) was measured with a total of three items “Most of my classmates are nice to me”, “I often take part in school/class activities”, and “I feel close to people in this school”. These items were measured on a four-point Likert scale (1 = Strongly disagree, 4 = Strongly agree). Cronbach’s alpha was 0.71.

#### 2.2.5. Covariates

This study used AP(T1), PA(T1), PPR(T1) in Grade 7 (Time1), depression (T2) in Grade 8 (Time 2), and parents’ education level and economic status were used as the control variables (covariates). Parents’ education level was coded as 1 if the parents did not receive education or graduated just from elementary, middle, or high school, and 2 if they graduated from college or higher. Economic status was measured with a question asking about current economic status (family circumstances) and was rated on a five-point Likert scale (1 = Very poor, 2 = Poor, 3 = Average, 4 = Rich, 5 = Very rich).

#### 2.2.6. Analysis Method

We checked whether the input variables satisfied the three assumptions of multiple regression analysis: 1. linearity of the phenomenon measured; 2. constant variance of the error terms; and 3. normality of the error term distribution. The scatter plot confirmed a linear relationship between the dependent and independent variables. The residual plot showed that the residuals were evenly distributed without a specific pattern; therefore, the assumption of constant variance of the error terms was satisfied. Finally, in the Q-Q plot, the error terms had a linear shape in the diagonal direction; therefore, the assumption of the normality of the error term distribution was also satisfied.

To analyze the research model, Model 11 and Model 18 provided by PROCESS [45] were used. To calculate the standardized regression coefficient, all input variables were converted into standardized scores for analysis. SPSS 26.0 was used for descriptive statistics, and the level of statistical significance was set as 0.05.

## 3. Results

### 3.1. Analysis of Basic Statistics

The correlations and descriptive statistics of the variables used in the analysis are shown in Table 1. The problem of multicollinearity was low because there was no high correlation between the variables and there was normal distribution with skewness ±3 or less and kurtosis ±7 or less [46].

### 3.2. Testing the Moderated Mediation Effect

PROCESS Model 18 was applied to test the research model in Figure 2. As shown in Table 2, after using SES, FEL, MEL, PD(T2), PPR(T1), AP(T1), and PA(T1) as covariates, PD(T1) had a statistically negative effect on PPR(T2) (β = −0.04, t = 3.05, *p* < 0.001), and R^2^ was statistically significant at 0.23 (F (8, 7161) = 261.61, *p* < 0.001). PPR(T2) had a statistically positive effect on AP(T2) (β = 0.04, t = 4.05, *p* < 0.001).

Depending on the interaction between PA(T2) as the primary moderator and gender as the secondary moderator, there was a statistically significant difference (a × b × c) in the impact of PPR(T2) on AP(T2) (β = −0.04 t = −2.15, *p* < 0.05). To analyze the effect of three-way interaction, this study divided the respondents with average physical activity—1 SD into the low activity group and those with + 1 SD into the high activity group and examined the difference in the indirect effect of PD(T1) → PPR(T2) → AP(T2) according to gender. Here, male students with low physical activity showed an indirect effect of −0.002 (95% CI (confidence interval) [−0.0041~−0.0004]) and female students showed −0.002 (95% CI [−0.0053~−0.0006]), thereby statistically significant since 95% CI did not include “0”. On the other hand, male students with high physical activity showed an indirect effect of −0.0015 (95% CI [−0.0036~−0.0001]) with 95% CI not including “0”, whereas female students showed 0.0006(95% CI [−0.0012~0.0027]) with 95% CI including “0”, thereby not showing statistical significance.

To summarize the study results, male students did not show a difference in the indirect effect of depression on academic performance through peer acceptance regardless of physical activity. However, for female students, more physical activity offset the indirect effect depression had on academic performance through peer acceptance. Therefore, for female students that participated in a lot of physical activity during adolescence, there was no indirect effect of depression on academic performance through peer acceptance, whereas for male students, there was an indirect effect regardless of physical activity. However, the moderated mediation effect of the research model in Figure 1 was not statistically significant.

## 4. Discussion

Depression can lead to a decline in academic performance as a result of decreased concentration on learning, decreased academic self-efficacy, and increased academic anxiety [15,16]. This study specifically examined the process in which experiencing depressive emotions in adolescence affects academic performance through longitudinal data.

As a result, higher depression in adolescence led to lower peer acceptance regardless of physical activity in male students, showing an indirect effect that led to decreased academic performance. On the other hand, there was no indirect effect with more physical activity in female students. In general, depression in adolescence leads to a decreased sense of belonging due to negative peer relationships, which may ultimately deteriorate academic performance with reduced learning motives and insincere learning attitudes [28]. Moreover, adolescents acknowledged by peers have greater needs to pursue academic goals with improved self-esteem [47,48]. Therefore, for male students, improving peer relationships is an important predictor variable for improving academic performance.

However, for female students who take part in a lot of physical activity, the indirect effect of depression on academic performance through peer acceptance was offset, implying that physical activity does not help improve the academic performance of female students. Rose and Rudolph [37] stated that female students have higher needs for affection and sensibility for interpersonal relations and value peer relationships more than male students. Thus, physical activity or sports participation does not help female students meet their needs for interpersonal relations, thereby showing a weaker social tie with peers. Since female students tend to be relatively easy-going and more rigorous than male students, are shy and introverted, and like quiet and dislike making noise, active activities such as sports participation do not help build extensive peer relationships [49]. Physical activity is a hindrance for female students to form a bond with peers. Thus, female students who participate in a lot of physical activity fail to build good peer relationships, which may lead to declined academic performance. It is not appropriate to conclude that physical activity does not help improve peer acceptance for female students only based on the results of this study. In general, physical education classes in China favor sports that are associated with masculinity, such as soccer and basketball. If male students practice yoga or Pilates, which are associated with femininity, will they gain increased popularity and attention among their peers? Thus, it is necessary to consider whether improvement in peer relationships is caused by physical and sports activities for female students, or by other social environmental factors. According to previous research, adolescents use physical activity as a helpful tool to improve peer relationships during the school transition (e.g., moving from middle school to high school) [50]. Although the developmental transition was not considered in this study, it is expected that female and male students who experience depressive symptoms during the school transition will demonstrate a positive effect of physical activity on academic performance than during the non-transition.

In this study, a moderated moderated-mediation effect did not exist in the research model of Figure 1. This indicates that, for Chinese adolescents participating in physical activity, depression had a negative effect on peer relationship regardless of gender. On the other hand, there was a moderated moderated-mediation effect in the research model of Figure 2. This indicates that physical activity and gender in the consecutive relationship of depression, peer relationship, and academic performance serve as moderated moderator variables in peer relationship and academic performance, not moderated moderator variables in the relationship between depression and peer relationship. For students experiencing depression, merely encouraging physical activity or sports participation does not help improve academic performance. Rather, participating in various sports programs to improve peer relationships may more positively impact academic performance.

In this study, Hypothesis 1 was partially accepted but Hypothesis 2 was rejected. One of the reasons is that gender identity regarding physical activity was not considered; only the physical activity of participants was measured. For example, can female students who are good at sports that are emphasized as masculine such as “soccer” and “basketball” be acknowledged and accepted by other female students? Conversely, will male students participating in activities that emphasize femininity such as “yoga” and “Pilates” be teased by other male students? As such, peer relationships may be affected by the gender identity of sports that students are participating in, thereby leading to the partial acceptance of Hypothesis 1 and rejection of Hypothesis 2. Therefore, for adolescents experiencing depressive emotions, it may be important to differentiate policies to promote physical activity according to gender to enhance policy efficiency.

This study expounds meaningful results by verifying the consecutive process of depression, peer relationships, and academic performance through panel data. The future research direction can be set as follows based on the limitations. First, it is necessary to conduct research on physical activity or sports emphasizing femininity. In this study, peer relationships did not help predict academic performance for female students who participate often in physical activity. During adolescence, both male and female students perceive gender-based social norms, with male students feeling the need to be macho and tough. Thus, when these gender-based social norms are violated, gender-role expectations induce negative responses from peers [51,52]. Therefore, female students participating in a lot of physical activity are included in the category of social norms emphasizing masculinity and thus may be rejected by peers. Future studies must examine how gender-role expectations actually affect peer relationships by examining sports that emphasize masculinity and femininity.

Second, we must consider omitted mediator variables. Since the direct effect of depression and academic performance was statistically significant in this study, this indicates that there are other mediator variables aside from peer relationships. The mediator variable that could theoretically be expected is procrastination. Procrastination is defined as the tendency to unnecessarily and intentionally put off a task or a decision [53]. Most people do not feel like doing anything when they are depressed, which may easily induce procrastination [54]. Therefore, adolescents experiencing depressive emotions show more procrastination about studying, which may ultimately lead to a decline in academic performance. Future studies must observe how the academic procrastination of adolescents with negative emotions is reduced by physical activity or sports participation.

Third, the present study used a self-reported survey to measure the research variables, so there is a possibility of social bias. Preliminary research on peer acceptance has used peer nominations to evaluate peer acceptance or rejection [51,55]. Hence, future research needs to examine the role of physical education activity in peer acceptance using a peer nomination technique to reduce social bias.

## 5. Conclusions

Adolescence is a transition period in which an individual spends most of their time with peers and moves on to adulthood by experiencing rapid physical and psychological changes. Depressive symptoms tend to increase in adolescence and academic performance, decreased by depression, is likely to lead to an interruption of study. The current study examined whether there is a difference in the consecutive process of depression to academic performance through peer relationships in adolescence according to the interaction between gender and physical activity. The results showed that, among female students with high depression experience, those who participated less in physical activity were more likely to show improvement in academic performance with improved peer relationships. For male students, academic performance was likely to improve due to improved peer relationships regardless of participation in physical activity.

## Figures and Tables

**Table 1:** Descriptive statistics and correlation analysis.

	1	2	3	4	5	6	7	8	9	10	11	12
1. SEX	-											
2. SES	−0.01	-										
3. FEL	0.03 *	0.22 ***	-									
4. MEL	0.02	0.20 ***	0.62 ***	-								
5. PD(T1)	0.02	−0.13 ***	−0.04 **	−0.04 ***	-							
6. PPR(T1)	0.08 ***	0.16 ***	0.11 ***	0.11 ***	−0.26 ***	-						
7. AP(T1)	0.25 ***	−0.01	0.06 ***	0.05 ***	−0.09 ***	0.18 ***	-					
8. PA(T1)	−0.10 **	0.06 **	0.05 **	0.04 **	−0.06 **	0.16 **	−0.03 **	-				
9. PD(T2)	0.05 ***	−0.08 ***	−0.02	−0.02	0.41 ***	−0.17 **	−0.04**	−0.02 *	-			
10. PPR(T2)	0.06 ***	0.12 ***	0.09 ***	0.09 ***	−0.22 ***	0.43 ***	0.11 ***	0.10 ***	−0.26 ***	-		
11. AP(T2)	0.20 ***	0.16 ***	0.23 ***	0.21 ***	−0.15 ***	0.26 ***	0.61 ***	−0.003	−0.08 ***	0.20 ***	-	
12. PA(T2)	−0.10 ***	0.11 ***	0.12 ***	0.12 ***	−0.06 ***	0.13 ***	0.02	0.15 ***	−0.05 ***	0.17 ***	0.15 ***	-
Mean	1.50	3.03	1.18	1.15	2.00	3.04	213.46	5.91	2.14	3.04	194.17	9.23
SD	0.50	0.55	0.38	0.35	0.8	0.72	23.55	6.31	0.92	0.69	57.8	6.35
Skewness	0.03	−0.11	1.75	2.04	1.07	−0.53	−0.92	1.59	0.91	−0.5	−0.75	1.53
Kurtosis	−2.00	2.87	1.07	2.15	1.61	−0.16	1.02	3.17	0.73	−0.13	−0.04	3.87

Note: SES = socioeconomic status; FEL = father’s education level; MEL = mother’s education level; PD = perceived depression; PPR = perceived peer relationship; AP = academic performance; PA = physical activities; * *p* < 0.05, ** *p* < 0.01, *** *p* < 0.001.

**Table 2:** Testing the moderated mediation effect of depression on academic performance.

		PPR(T2)	AP(T2)
		β	β
COV	SES	0.04 ***	0.09 ***
	FEL	0.03 *	0.10 ***
	MEL	0.01	0.10 ***
	PPR(T1)	0.36 ***	0.08 ***
	PD(T2)	−0.17 **	0.001
	AP(T1)	0.04 ***	0.57 ***
	PA(T1)	0.06 ***	−0.01
MOV	Sex ^c^		0.12 ***
	PA(T2) ^b^		0.02 ***
IV	PD(T1)	−0.04 **	−0.05 ***
	PPR(T2) ^a^		0.04 ***
	a × b		−0.03 **
	a × c		−0.02
	b × c		0.06 **
IT	a × b × c		−0.04 *
	R	0.48	0.68
	R^2^	0.23	0.46
		F (8, 7161) = 261.61 ***	F (15, 7154) = 414.25 ***

Note: Coded as male = 1, female = 2; MV = mediator variable; DV = dependent variable; IV = independent variable; COV = covariate; MOV = moderator variable; IT = interaction term; SES = socioeconomic status; FEL = father’s education level; MEL = mother’s education level; PD = perceived depression; PPR = perceived peer relationship; AP = academic performance; PA = physical activities. * *p* < 0.05, ** *p* < 0.01, *** *p* < 0.001. a = PPR(T2), b = PA(T2), c = Sex.

## Data Availability

Not applicable.
